# Genetic and Small-Molecule Modulation of Stat3 in a Mouse Model of Crohn’s Disease

**DOI:** 10.3390/jcm11237020

**Published:** 2022-11-28

**Authors:** Prema Robinson, Emily Magness, Kelsey Montoya, Nikita Engineer, Thomas K. Eckols, Emma Rodriguez, David J. Tweardy

**Affiliations:** 1 Departments of Infectious Diseases, Infection Control & Employee Health, The University of Texas MD Anderson Cancer Center, Houston, TX 77030-3772, USA; 2 Molecular & Cellular Oncology, Division of Internal Medicine, The University of Texas MD Anderson Cancer Center, Houston, TX 77030-3772, USA

**Keywords:** STAT3, inflammatory bowel disease, Crohn’s disease

## Abstract

Crohn’s disease (CD), is an inflammatory bowel disease that can affect any part of the gastro-intestinal tract (GI) and is associated with an increased risk of gastro-intestinal cancer. In the current study, we determined the role of genetic and small-molecule modulation of STAT3 in a mouse model of CD. STAT3 has 2 isoforms (α, β) which are expressed in most cells in a 4:1 ratio (α: β). STAT3α has pro-inflammatory and anti-apoptotic functions, while STAT3β has contrasting roles. We used an animal model of CD consisting of intrarectal administration of 2,4,6-trinitrobenzene sulfonic acid and examined the severity of CD in transgenic-mice that express only STAT3α (∆^β^/∆^β^), as well as in wild-type (WT) mice administered TTI-101 (formerly C188-9), a small molecule STAT3 inhibitor. We determined that clinical manifestations of CD, such as mortality, rectal-bleeding, colonic bleeding, diarrhea, and colon shortening, were exacerbated in ∆^β^/∆^β^ transgenic versus cage-control WT mice, while they were markedly decreased by TTI-101 treatment of WT mice. TTI-101 treatment also increased apoptosis of pathogenic CD4^+^ T cells and reduced colon levels of IL-17-positive cells. Our results indicate that STAT3 contributes to CD and that targeting of STAT3 with TTI-101 may be a useful approach to treating CD.

## 1. Introduction

Inflammatory bowel disease (IBD) is a chronic relapsing inflammatory disorder that affects the gastro-intestinal tract [1]. Ulcerative colitis (UC) and Crohn’s disease (CD), the two major forms of IBD are distinguished by the location of the inflammation within the gastro-intestinal tract. Different from UC where the inflammation typically involves the rectum and extends up the colon for a variable extent affecting only the superficial mucosa; CD can affect any part of the gastro-intestinal tract, although the distal ileum and colon are the parts most often affected [2,3]. Symptoms of the disease includes one or more of the following; abdominal pain, clinical signs of bowel obstruction or diarrhea with passage of blood or mucus, or both, delayed development and stunted growth in children, fever, rectal bleeding and weight loss [1,4,5]. IBD affects approximately 1.6 million Americans, this figure includes people with Crohn’s disease and ulcerative colitis [6]. CD is associated with an increased risk of colorectal cancer [7,8].

The pathogenesis of Crohn’s has been linked to microbial, immunological, and environmental factors [2,9,10]. There is strong evidence that a genetic disposition is a major key in acquiring CD [11]. A large number of genetic variants identified in Crohn’s disease are related to genes targeting microbial recognition and bacterial wall sensing. CD susceptibility genes include autophagy genes (*ATG16L1* and *IRGM)* [2], and genes involved in both the innate (*TLR4*, *CARD9*, *IL23R*, *STAT3*) and adaptive immune system (*HLA*, *TNFSF15*, *IRF5*, *PTPN22*) pathways [12,13,14,15,16,17,18]. These genes implicate the role of antimicrobial peptides, innate and adaptive immune cell function, and cytokines (tumor necrosis factor and interleukins 6, 12, 17, 22, and 23) in pathogenesis of CD. Several of the above-mentioned cytokines serve as ligands for cell surface receptors that activate signal transducer and activator of transcription (STAT) 3. STAT3 is a key transcriptional regulator that controls cell growth, programmed cell death pathways, and inflammation in response to intrinsic and environmental stimuli. STAT3 has been found to contribute, with contrasting effects, to colitis in three cell lineages (myeloid cells, enterocytes, and T cells) in mice and humans [19,20,21,22]. Genetic deletion of STAT3 in mouse myeloid cells (neutrophils and macrophages) resulted in chronic colitis due to increased Th1 and decreased IL-10 responses. Genetic deletion of STAT3 in enterocytes rendered mice more susceptible to experimental colitis [23]. Hence, STAT3 in these two cell lineages was protective against colitis. Meanwhile, studies in mice demonstrated that STAT3 prevents apoptosis of infiltrating pathogenic T cells, resulting in chronic intestinal inflammation [24], which indicated that STAT3 activation in T cells contributes to colitis. There have been no studies to date that examine the net contribution of STAT3 in all three cell populations to CD.

STAT3 exists in the form of two isoforms, α (p92) and β (p83), derived from a single gene through alternative mRNA splicing. These isoforms are expressed in most cells in a 4:1 ratio (α: β) [25,26,27]. Studies in mice deficient in one or both STAT3 isoforms delineate distinct functions for STAT3, as well as for each isoform, in development, inflammation, and apoptosis [28,29,30,31,32,33]. STAT3α is endowed with pro-inflammatory and anti-apoptotic function, while STAT3β opposes the effects mediated by STAT3α [28,29,30,31,32,33].

2,4,6-trinitrobenzene sulfonic acid (TNBS)-induced CD is a commonly used animal model of CD that shares significant properties with human Crohn’s disease [34]. This model involves one-time intrarectal administration of 2,4,6-trinitrobenzenesulfonic acid (TNBS) and has been used to study disease pathogenesis and pre-clinical testing of novel drugs [35,36].

Crohn’s disease can be controlled but remains incurable. The mainstay therapies for CD include anti-inflammatory drugs and immunomodulatory agents such as enteric-coated budesonide or prednisolone, anti-TNFα inhibitors [37]. However, these interventions are often unsatisfactory as relapses often occur. Although considerable progress has been made over the past two-to-three decades to treat this debilitating disease, more effective therapies are needed.

In this study, we will determine the contribution of STAT3 to CD induced by TNBS administration by examining mice that express only STAT3α. We will further determine the efficacy ofTTI-101, a small molecule inhibitor of both isoforms of STAT3 that is entering Phase II studies in cancer in CD in WT mice. These studies will provide proof-of-concept small-molecule targeting of STAT3 is worthy of consideration as a novel approach to CD treatment.

## 2. Materials and Methods

### 2.1. TNBS Models of CD

In one set of studies, 8-week-old STAT3α knock-in/STAT3β-deficient ∆^β^/∆^β^ mice and cage control wild type mice were randomly assigned for induction of TNBS-induced CD. Acute TNBS-induced CD in mice was induced by a one-time intrarectal (IR) administration of 100 mg/kg TNBS in 50% ethanol. At 48 h post administration of TNBS, mice were euthanized, and intestinal tissues were harvested. The administration of ethanol is a prerequisite to break the colonic mucosal barrier to allow penetration of TNBS into the lamina propria. Rodents were maintained for few minutes at a head down position in order to avoid expulsion of the fluid and ensure even distribution of the chemical [34].

Founder ∆^β^/∆^β^ mice were kindly provided by Dr. Valerie Poli, Department of Genetics, Biology, and Biochemistry, University of Turin, Italy [29] and were re-derived in a BALB/C background. In the second set of studies, the impact of TTI-101 was examined, 8 week old BALB/C mice (Envigo, Indianapolis, IN, USA), were randomly assigned to one of four groups: (1) one time rectal administration of TNBS (100 mg/kg in ethanol) plus TTI-101 [100 mg/kg body weight in DMSO (100 μL) administered intraperitoneally (IP) 30 min prior to TNBS and once 24 h later], (2) one time rectal administration of TNBS plus DMSO (100 μL) (IP) 30 min prior to TNBS and once 24 h later (3) ethanol (IR) plus TTI-101(IP) as above, and (4) Ethanol (IR) plus DMSO (IP) as above. We obtained TTI-101, a small molecule inhibitor of STAT3 developed by Tvardi Therapeutic, Inc., and used it as described previously [38,39,40,41,42,43]. Forty-eight post TNBS/ethanol administration, mice were evaluated for rectal bleeding, colonic bleeding and stool consistency. Rectal bleeding and colonic bleeding were assessed visually and graded (scale of 0 to 3). Stool consistency was graded (scale of 0 to 3, where: 0 = normal, 1 = soft, 2 = very soft, and 3 = diarrhea). After the mice were euthanized, their colons were harvested, length measured and a portion was fixed in 10% formalin, embedded in paraffin (FFPE), sectioned and stained with hematoxylin and eosin (H&E). The H&E stained slides were then examined microscopically and scored (0 to 3+) based on the extent of inflammatory cell infiltration. Scores for rectal bleeding, stool consistency, and inflammation are presented as mean score ± SD for each group. Duplicate experiments were performed, and results are expressed as mean ± SEM unless otherwise stated. Significant differences for each of the assessment scores and colon length differences are indicated (using ANOVA followed by Tukey’s as a post-test).

### 2.2. Immunohistochemical and TUNEL Analysis

Transverse sections of formalin-fixed paraffin-imbedded (FFPE) colon were examined using immunohistochemistry (IHC) for CD4 and IL-17A staining, as described [44]. We stained adjacent sections for control staining and TUNEL staining, as previously described [45]. For IHC, slides were incubated with polyclonal rabbit antibody against CD4 or IL-17A (cat #BS 0647-R and BS 1183-R, Bioss, Woburn, MA, USA) at dilutions of 1/250 and 1/1000, respectively, and developed using the automated bond polymer refine detection kit (cat #DS 9800, Leica Biosystems, Buffalo Grove, IL, USA). Adjacent sections stained with polyclonal control rabbit antibody were included as controls. To avoid bias, light microscopic examination was performed in a blinded fashion by assessors who were not aware of outcomes nor the groups that the mice were allocated to. The number of positive brown cells with intensity above background control were counted at 400× in 20–30 fields. TUNEL assay was performed using the ApopTag plus Peroxidase in situ Apoptosis Detection Kit (Chemicon) according to the manufacturer’s instructions, as described [45]. The percentage of CD4^+^ cells undergoing apoptosis was determined by assessing 500–1000 CD4^+^ cells in the CD4 stained slide and calculating the percentage of these cells that were TUNEL-positive. Data presented are the mean percentage ± SEM for each group; significant differences are indicated (using ANOVA, followed by Tukey’s as a post-test).

### 2.3. Measurements of Y705-Phosphorylated (pY) STAT3 and Total STAT3 Using Luminex Bead-Based Assays

We determined levels of Y705-phosphorylated (*p*) STAT3 and total STAT3 by the Luminex bead technology. Flash frozen tissue sections were homogenized in lysis buffer and assayed for pY-STAT3 and total STAT3 using reagents provided in the Millipore (Milliplex) kits, as described by the manufacturer. Levels of each protein analyte were determined and analyzed using the Bio-Plex suspension array system (Bio-Rad). The total and pY-STAT3 levels were normalized to total protein levels. Total protein was quantitated using the Bradford method (cat #500-0006, Bio-Rad, Hercules, CA, USA).

### 2.4. Statistical Analyses

Statistical differences in endpoints such as diarrheal score, colonic bleeding score, colon length, apoptosis of CD4 cells, pY-STAT3 levels, colonic inflammatory score and Th17 levels between groups were determined using one-way ANOVA, followed by a pairwise comparison using Tukey’s. Percent survival between groups was statistically compared using Kaplan–Meier analysis. Significance was set at *p* < 0.05.

## 3. Results

### 3.1. Effect of STAT3β Deletion on TNBS-Induced CD in Mice

**Figure 1:**
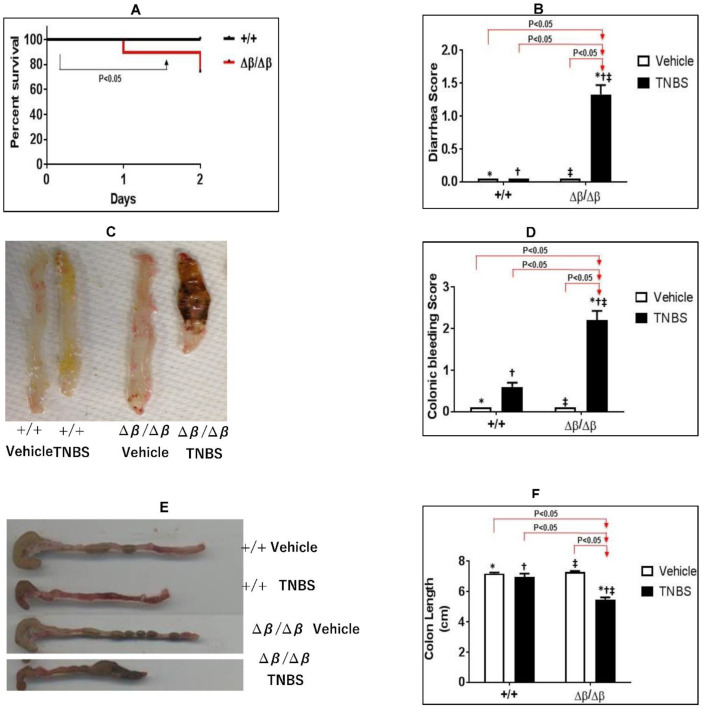
**Effects of TNBS-induced CD on mortality, diarrhea, colonic bleeding, and colon shortening in +/+ versus** ∆^β^/∆^β^ **mice**. Effect of STAT3 deletion on TNBS-induced CD was studied in +/+ and ∆^β^/∆^β^ mice. (**A**) Survival was assessed daily and was 74% in the TNBS ∆^β^/∆^β^ mice group vs. 100% in the TNBS +/+ mice group 48 hrs. post-administration of TNBS (*p* < 0.05, Kaplan–Meier analysis, *n* = 9–19). (**B**) Diarrheal score, (**C**,**D**) colonic bleeding, (**E**,**F**), and colon length were assessed 48 hrs. post-administration of TNBS. Results are expressed as the mean ± SEM of two separate experiments [*, vehicle (+/+ mice) vs. TNBS (∆^β^∆^β^/mice)], [^†^, TNBS (+/+ mice) vs. TNBS (∆^β^∆^β^/mice)], and [^‡^, vehicle (∆^β^/mice) vs. TNBS (∆^β^∆^β^/mice)]; all *p* < 0.05, ANOVA followed by Tukey’s post-test; *n* = 6–12.

To determine the effect of STAT3β deletion and the resultant unopposed action of STAT3α on TNBS-induced CD in mice, we examined mortality, weight-loss, rectal bleeding, colonic bleeding, diarrhea, colon shortening, and apoptosis of CD4^+^ T-cells in transgenic STAT3α knock-in/STAT3β-deficient (∆^β^/∆^β^ mice and in wild type littermate cage control (+/+) mice administered TNBS intrarectally (Figure 1). The experiment was terminated 48hrs post administration of TNBS. Mortality was 26% in ∆^β^/∆^β^ mice given TNBS compared to 0% in +/+ mice (*p* < 0.05, Kaplan–Meier analysis; *n* = 19; Figure 1A). There was no significant weight loss seen in TNBS-treated, ∆^β^/∆^β^ or in the +/+ mice groups compared to their respective control non-TNBS treated groups. (*p* > 0.05, Student’s unpaired t-test; results not shown). Diarrheal scores in ∆^β^/∆^β^ mice (1.33 ± 0.1420) were significantly higher than in +/+ mice (0 ± 0; *p* < 0.05, ANOVA followed by a pair-wise comparison with Tukey’s test, *n* = 6–12; Figure 1B). Colonic bleeding scores in ∆^β^/∆^β^ mice given TNBS (2.208 ± 0.258) were also significantly higher than in +/+ mice (0.6 ± 0.1; *p* < 0.05, ANOVA followed by a pair-wise comparison with Tukey’s test, *n* = 6–12; Figure 1C,D). Similarly, rectal bleeding scores in ∆^β^/∆^β^ mice given TNBS (2.083 ± 0.1930) were significantly higher than in +/+ mice (1.20 ± 0.20; *p* < 0.05, ANOVA followed by a pair-wise comparison with Tukey’s test, *n* = 6–12; results not shown). Furthermore severity in clinical manifestations of TNBS-induced CD in ∆^β^/∆^β^ mice was accompanied by marked shortening of the colon; Figure 1E), which was 21% shorter in ∆^β^/∆^β^ mice (54.92 ± 1.228 cm) than in +/+ mice (69.80 ± 2.177 cm; *p* < 0.05, ANOVA followed by a pair-wise comparison with Tukey’s test, *n* = 6–12; Figure 1F).

### 3.2. Effect of TNBS-Induced CD on Apoptosis of CD4^+^ Cells in Colons of +/+ and ∆^β^/∆^β^ Mice

**Figure 2:**
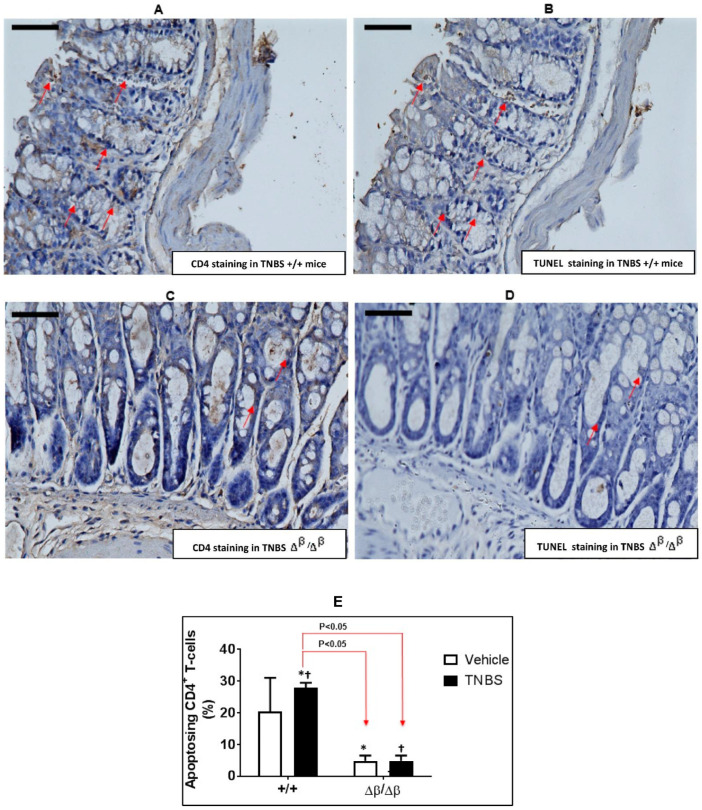
**Effect of TNBS-induced CD on apoptosis of CD4^+^ cells in colons of** +/+ **and** ∆^β^/∆^β^ **mice.** Representative photomicrographs of CD4-stained and TUNEL-stained sections of colons (magnification 400×) derived from; (**A**,**B**) TNBS +/+ mice and (**C**,**D**) TNBS ∆^β^/∆^β^ mice. Red arrows depict cells positively stained for CD4 or TUNEL. Scale bar on each micrograph represents 50 microns. CD4^+^ cells (500–1000 per section) within colon sections IHC stained for CD4 were assessed on the adjacent slide for TUNEL positivity or negativity. (**E**) The percentage TUNEL-positive cells expressed as mean ± SD ((*, TNBS (+/+ mice) vs. vehicle (∆^β^/∆^β^ mice), ^†^, TNBS (+/+ mice) vs. TNBS (∆^β^/∆^β^ mice), both *p* < 0.05, ANOVA followed by Tukey’s post-test; *n* = 3–4).

In order to determine if reduced apoptosis within pathogenic CD4^+^ T cell is responsible for the worsening of TNBS-induced CD observed in ∆^β^/∆^β^ mice, we evaluated the colons of mice and determined the percent of CD4^+^ T cells that underwent apoptosis (Figure 2). There was a 82.75% decrease in the percentage of CD4^+^ T-cells undergoing apoptosis in response to TNBS in the colons of ∆^β^/∆^β^ mice (4.8% ± 1.72%) compared to +/+ mice (27.84% ± 1.61%; Figure 2, *p* < 0.05, ANOVA followed by a pair-wise comparison with Tukey’s test, *n* = 2–4; Figure 2). Accordingly, the severity of TNBS-induced CD in ∆^β^/∆^β^ mice may be due, in part, to enhanced survival of CD4^+^ T cells. This suggests, further, that the contribution of unopposed STAT3α to TNBS-induced colitis in CD4^+^ cells supersedes its effects in myeloid and epithelial cells.

### 3.3. Effects of TTI-101 Treatment on TNBS-Induced CD

**Figure 3:**
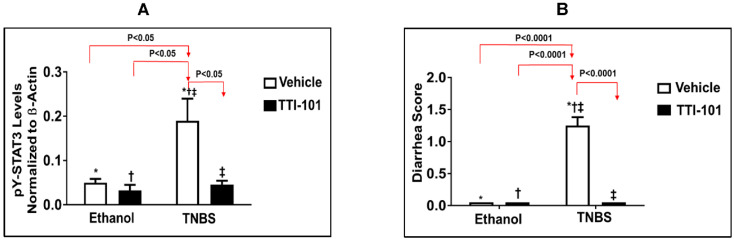

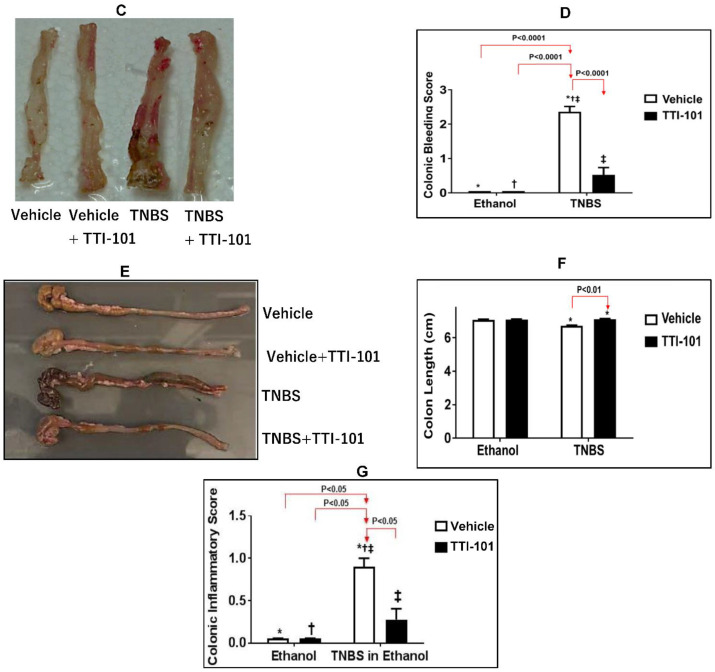
**Effect of TTI-101 treatment on colon levels of pY-STAT3 protein, diarrhea, colonic bleeding, colon length, and colon inflammation in TNBS-induced CD.** (**A**) pY-STAT3 levels were measured by Luminex assay in the colons of mice that received 50% ethanol or TNBS in 50% ethanol and treated with vehicle (DMSO) or TTI-101 in DMSO. pY-STAT3 levels were normalized to β-tubulin protein levels and the means ± SD of the ratios plotted. (**B**) Diarrheal score, (**C**,**D**) colonic bleeding, (**E**,**F**), colonic inflammation, and (**G**) colon length were assessed 48 hrs. post-administration of TNBS. Results are expressed as the mean ± SEM of two separate experiments ((**A**,**B**,**D**,**G**): *, Ethanol vs. TNBS, ^†^, Ethanol+TTI-101 vs. TNBS, and ^‡^, TNBS vs. TNBS+TTI-101, all *p* < 0.05; ANOVA followed by Tukey’s post-test; *n* = 3–12), (**F**): *, TNBS vs. TNBS+TTI-101. *p* < 0.05; ANOVA followed by Tukey’s post-test; *n* = 6–12).

To further examine the involvement of STAT3 across all tissues and cells to TNBS-induced CD, we examined the effect of TTI-101, a small molecule inhibitor of STAT3 developed by Tvardi Inc., on TNBS-induced CD in mice. TTI-101 targets the Src-homology (SH) 2 domain of STAT3, which inhibits two steps in its activation—recruitment to activated receptor complexes and homodimerization [46]. TTI-101 has been demonstrated to successfully target STAT3 in several mouse models of inflammation [42,43,47,48,49] and cancer [38,41,50,51]. We demonstrated that levels of pY-STAT3 were increased in the colons of mice administered TNBS (*p* < 0.05, ANOVA followed by a pair-wise comparison with Tukey’s test, *n* = 3–6; Figure 3A). However, there was no significant difference in the levels of total STAT3 (results not shown). Importantly, we demonstrated that TTI-101 treatment reduced levels of pY-STAT3 by 76% compared to that vehicle-treated TNBS mice and normalized them (*p* < 0.05, ANOVA followed by a pair-wise comparison with Tukey’s test, *n* = 3–6; Figure 3A). The administration of TTI-101 to TNBS mice also: (1) normalized scores for diarrhea and colonic bleeding (*p* < 0.05 for both, ANOVA followed by a pair-wise comparison with Tukey’s test; *n* = 6–12; Figure 3C,D), (2), normalized colon length (*p* < 0.05, *n* = 6–12; Figure 3E,F), and (3) reduced colonic inflammation to levels indistinguishable from controls (*p* < 0.05, ANOVA followed by a pair-wise comparison with Tukey’s test; *n* = 3–5; Figure 3G).

### 3.4. Effect of TTI-101 Treatment on Apoptosis of CD4^+^ Cells

**Figure 4:**
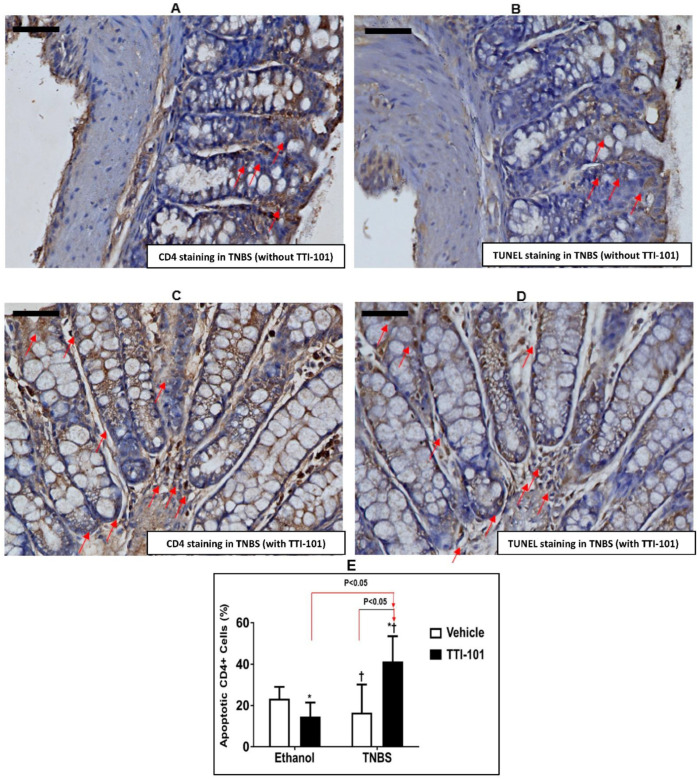
**Effect of TTI-101 treatment on TNBS-induced apoptosis in CD4 cells.** (**A**,**B**) Photomicrographs of CD4 and TUNEL stained sections of colon tissue derived from TNBS mice without TTI-101 treatment and from (**C**,**D**) TNBS mice with TTI-101 treatment, respectively (magnification 400×). Red arrows depict cells positively stained for CD4 or TUNEL. Scale bar on each micrograph represents 50 microns. (**E**) Percentage of apoptosis in colonic CD4+cells in response to TNBS, with and without TTI-101 treatment. Results are expressed as mean ± SD (*, Ethanol+TTI-101 vs. TNBS and ^†^, TNBS vs. TNBS+TTI-101; all *p* < 0.05, ANOVA followed by Tukey’s post-test; *n* = 3–4).

Consistent with the results obtained in the ∆^β^/∆^β^ mice, treatment with TTI-101 increased the percentage of CD4^+^ T-cells undergoing apoptosis in response to TNBS (41.39 ± 12.06%) compared to untreated mice (16.55 ± 13.61%; *p* < 0.05, ANOVA followed by a pair-wise comparison with Tukey’s test, *n* = 3–4; Figure 4). According to these findings, STAT3α activity within CD4^+^ cells in the absence of opposing pro-apoptotic effects of STAT3β or following treatment with TTI-101, results in greater survival CD4^+^ T-cells, which leads to worse manifestations of TNBS-induced CD.

### 3.5. Effect of TTI-101 Treatment on Colonic Th17 Cell Infiltration

**Figure 5:**
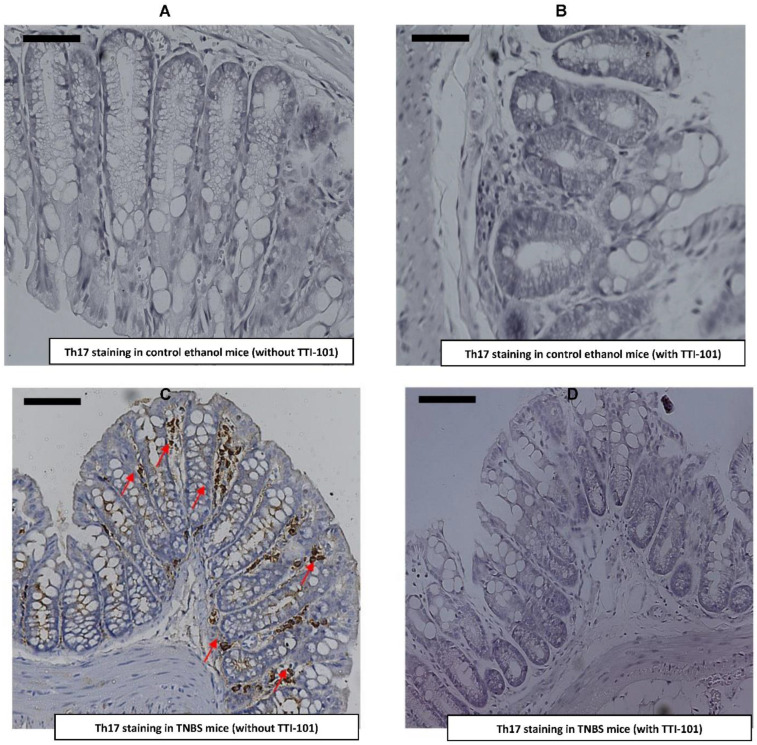

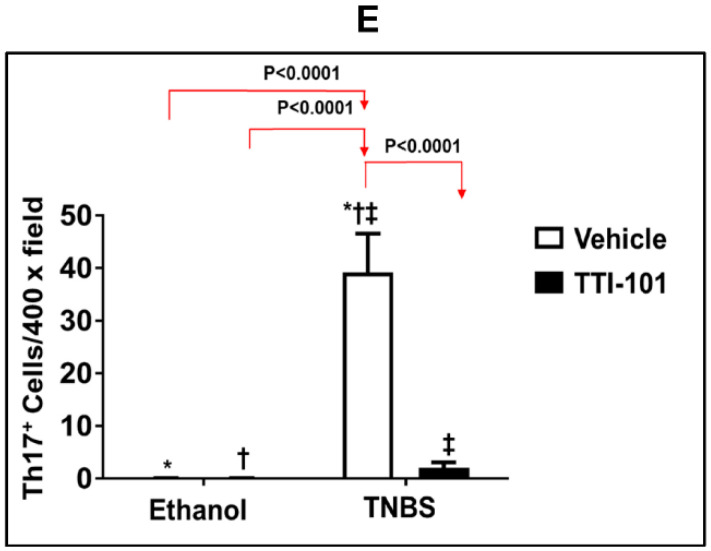
**Effect of TTI-101 treatment on colonic Th17 cell infiltration.** (**A**) Photomicrographs of Th17-stained sections of colons derived from control mice without TTI-101 treatment or (**B**) control mice with TTI-101 treatment or (**C**) TNBS mice without TTI-101 treatment, and (**D**) TNBS mice with TTI-101 treatment (magnification 400×). Red arrows depict cells positively stained for Th17. Scale bar on each micrograph represents 50 microns. (**E**) TTI-101 treatment significantly decreased levels of TNBS-induced colonic Th17 cell infiltration. Results are expressed as the mean ± SEM (average Th17 number in *, Ethanol vs. TNBS, ^†^, Ethanol+TTI-101 vs. TNBS and ^‡^, TNBS vs. TNBS+TTI-101, all, *p* < 0.05, ANOVA followed by Tukey’s post-test) (*n* = 3–4).

Genome-wide association studies have revealed that *IL23R* and five additional genes involved in Th17 differentiation (*IL12B*, *JAK2*, *STAT3*, *CCR6* and *TNFSF15*) are associated with susceptibility to Crohn’s disease [52]. Several studies have demonstrated that Th17 cells may play an important role in intestinal inflammation in Crohn’s disease [52]. Additionally, CD4^+^Th17^+^ cells have been shown to depend on STAT3 for their normal development [53]. In order to determine whether TTI-101 treatment affected the number of IL-17-producing cells induced by TNBS within the colon, we determined levels of IL-17-positive cells by immunohistochemistry within the colon of mice administered TNBS without or with TTI-101 treatment. TNBS administration markedly increased the number of IL-17-positive cells in the colon (39.2 ± 7.35) compared to mice not given TNBS (0 ± 0; *p* < 0.05, ANOVA followed by a pair-wise comparison with Tukey’s test; *n* = 2–5; Figure 5). TTI-101 treatment decreased the number of IL-17-positive cell in the colon (2 ± 1.08) by 95% (*p* < 0.05, ANOVA followed by a pair-wise comparison with Tukey’s test; *n* = 2–5; Figure 5).

## 4. Discussion

We evaluated the effect on CD of increased STAT3 pro-inflammatory and anti-apoptotic activity within all cells (enterocytes, myeloid cells, and T cells) implicated in its pathogenesis. We enrolled mice that globally expressed only the pro-inflammatory and anti-apoptotic isoform of STAT, Stat3α (Stat3β knockout mice), into the TNBS mouse model of CD. As reported, administration of TNBS to WT mice caused shortening of the colon, loose stools, and rectal bleeding. Of note, each of these manifestations of colitis was exacerbated in Stat3β knockout mice and was accompanied by mortality. Apoptosis of CD4^+^ T cells, but not of myeloid cells or enterocytes, was readily detected in the colons of TNBS-treated WT mice; importantly, their frequency was markedly reduced in TNBS-treated Stat3β knockout mice vs. wild type mice, which helps to explain the increased severity of disease. To complement these findings and to determine if systemic targeting of STAT3 may be of benefit in CD, we treated WT mice that received TNBS with either vehicle control or a small-molecule STAT3 inhibitor, TTI-101. Administration of TTI-101 to TNBS-treated mice prevented all CD manifestations and normalized colon levels of pY-STAT3. These findings suggest that the net effect of STAT3 across all cells and tissues is to promote the development of CD. Our results also provide proof-of-concept that systemic targeting of STAT3 with TTI-101, which is entering Phase II clinical trials for the treatment of solid tumors, has the potential to be developed into a new treatment for CD.

Studies by other groups have shown that the precise role of STAT3 in pathogenesis of IBD may be cell specific. STAT3 within enterocytes and myeloid cells is protective in IBD, while STAT3 within T-cells is detrimental to pathogenesis of IBD. For example, genetic deletion of STAT3 in mouse myeloid cells (neutrophils and macrophages) or in enterocytes resulted in increased severity and susceptibility to experimental colitis [19,23,54] suggesting that STAT3 in these two cell lineages was protective against IBD. In contrast, other studies in mice demonstrated that STAT3 prevents apoptosis of infiltrating pathogenic T cells, which results in chronic intestinal inflammation [20,21,22,24], indicating that STAT3 activation in T cells is detrimental and contributes to IBD. The results of the current studies suggest that the effects of STAT3 modulation within the T cell compartment is dominant over the effects of STAT3 modulation in the other two cell compartments.

Genetic studies have implicated *IL23R* and five additional genes that are involved in Th17 differentiation (*IL12B*, *JAK2*, *STAT3*, *CCR6* and *TNFSF15*) in susceptibility to CD [52]. We demonstrated that the levels of Th17 cells are increased in colons in response to TNBS-induced CD, and, importantly, that the influx of Th17 into the colons of TNBS-treated mice is markedly reduced by treatment with TTI-101.

## 5. Conclusions

These findings support the conclusion that STAT3, particularly within CD4^+^ T cells, contributes to the pathogenesis of CD and provides proof-of-concept that targeting of STAT3 with a small molecule may serve as a new approach for treatment of CD.

## Data Availability

Not applicable.
